# Osteoarthritis-dependent changes in antinociceptive action of Na_v_1.7 and Na_v_1.8 sodium channel blockers: An *in vivo* electrophysiological study in the rat

**DOI:** 10.1016/j.neuroscience.2015.03.042

**Published:** 2015-06-04

**Authors:** W. Rahman, A.H. Dickenson

**Affiliations:** Department of Neuroscience, Physiology and Pharmacology, University College London, Gower Street, London WC1E 6BT, UK

**Keywords:** C-LTMs, C-low-threshold mechanoreceptors, DRG, dorsal root ganglia, KO, knockout, MIA, monosodium iodoacetate, NSAIDs, non-steroidal anti-inflammatory drugs, OA, osteoarthritis, PWF, paw withdrawal frequency, PWT, paw withdrawal threshold, RM ANOVA, repeated-measures analysis of variance, SEM, standard error of the mean, vF, von Frey, VGSCs, voltage-gated sodium channels, WDR, wide dynamic range, osteoarthritis, pain, dorsal horn, sodium channels, electrophysiology

## Abstract

Voltage-gated sodium channel blockers are not traditionally recommended for osteoarthritis (OA) pain therapy, but given the large peripheral drive that follows OA development there is a rationale for their use. Using a rat model of monosodium iodoacetate (MIA)-induced OA we used *in vivo* electrophysiology to assess the effects of the Na_v_1.7- and Na_v_1.8-selective antagonists, ProTxII and A-803467 respectively, on the evoked activity of spinal dorsal horn neurons in response to electrical, mechanical and thermal stimuli applied to the peripheral receptive field. These studies allow examination of the roles of these channels in suprathreshold stimuli, not amenable to behavioral threshold measures. Spinal administration of ProTxII significantly reduced neuronal responses evoked by mechanical punctate (von Frey (vF) 8–60 g) and noxious thermal (45 and 48 °C) stimuli in MIA rats only. A-803467 significantly inhibited neuronal responses evoked by vF 8–60 g and 48 °C heat after spinal administration; significantly inhibited responses evoked by brush, vFs 26–60 g and 40–48 °C stimuli after systemic administration; significantly inhibited the electrically evoked Aδ-, C-fiber, post-discharge, Input and wind-up responses and the brush, vFs 8–60 g and 45–48 °C evoked neuronal responses after intra plantar injection in the MIA group. In comparison A-803467 effects in the sham group were minimal and included a reduction of the neuronal response evoked by vF 60 g and 45 °C heat stimulation after spinal administration, no effect after systemic administration and an inhibition of the evoked response to 45 °C heat after intra plantar injection only. The observed selective inhibitory effect of ProTxII and A-803467 for the MIA-treated group suggests an increased role of Na_v_1.7 and 1.8 within nociceptive pathways in the arthritic condition, located at peripheral and central sites. These findings demonstrate the importance of, and add to, the mechanistic understanding of these channels in osteoarthritic pain.

## Highlights

• MIA-dependent antinociceptive effect of ProTxII and A-803467 on neuronal activity.
• Changes in Na_v_1.7 and 1.8 channel function contribute to osteoarthritic pain.
• Blocking Na_v_1.7 and Na_v_1.8 channels has therapeutic potential for the treatment of osteoarthritic pain.

## Introduction

Osteoarthritis (OA) constitutes one of the largest cost burdens to healthcare in the western world with pain being the dominant symptom and reason for clinical presentation (Hiligsmann et al., 2013; Neogi, 2013). Non-steroidal anti-inflammatory drugs (NSAIDs) are first-line treatments, often in combination with paracetamol or opioids, but analgesic efficacy is largely modest at best at tolerable doses, or is hampered by significant adverse effects with dose escalation (Harvey and Hunter, 2010; Zhang et al., 2010a). For these reasons, many patients resort to total joint replacement to relieve their pain, yet chronic pain remains for a significant proportion (about 20–40%) of patients (Kirwan et al., 1994; Creamer et al., 1996; Ethgen et al., 2004). This highlights the complexity of OA pain and the significant unmet clinical need.

OA is characterized by inflammation (episodic and chronic) and swelling of joints and also significant pain in the area surrounding the joint and often in areas distant to the affected joint (referred pain), thus suggesting that both peripheral and central nociceptive mechanisms are at play (Farrell et al., 2000; Malfait and Schnitzer, 2013; Zhang et al., 2013). The transmission of pain from the peripheral site of injury, beyond the peripheral transducers, requires activation of voltage-gated sodium channels (VGSCs) located on peripheral nociceptors. Abundant data exist showing that maladaptive changes in VGSCs are critical for mediating variety of chronic pain conditions in both animals and humans (Eijkelkamp et al., 2012; Dib-Hajj et al., 2013) thus modulating their activity is a rational strategy for chronic pain therapy.

Sodium channel blockers for the treatment of OA pain are not currently recommended, yet they may have a key role in controlling OA pain since there is strong evidence for abnormal firing in peripheral and central neurons in the arthritic condition, which must involve alterations in VGSCs (Schuelert and McDougall, 2006, 2008, 2009; McDougall et al., 2009; Rahman et al., 2009; Sagar et al., 2010; Kelly et al., 2012, 2015; Bullock et al., 2014) and a genetic mutation in the encoding gene for the 1.7 sodium channel sub-type has been correlated with increased pain sensitivity in OA patients (Reimann et al., 2010) but see (Valdes et al., 2011). Furthermore, analgesic efficacy of the lidocaine patch and intravenous and intra-articular injection of non-selective VGSC blockers has been observed in osteoarthritic patients (Creamer et al., 1996; Burch et al., 2004; Gammaitoni et al., 2004; Kivitz et al., 2008; Dworkin et al., 2011; Duarte et al., 2014).

There is a rationale for sodium channel blockers for OA pain therapy, based on heightened peripheral drive, which could be present in both early inflammatory and later non-inflammatory stages. In addition there may be neuropathic components to the pain in sub-groups of patients (Duarte et al., 2014; Thakur et al., 2014). Our aim was to further characterize the role of Na_v_1.7 and 1.8 channels in a rat model of monosodium iodoacetate (MIA) (2 mg)-induced OA of the knee joint; a well-established model for the mechanistic study of osteoarthritic pain that has also been pharmacologically validated with respect to established analgesics including NSAIDs (Vonsy et al., 2008; Zhang et al., 2013). This dose of MIA (2 mg) has been shown to produce an up-regulation of the neuronal damage marker, cAMP-dependent transcription factor (ATF-3), in peripheral nerves that innervate the knee joint, a reduction in intra-epidermal nerve fiber density and alterations in spinal cord neuroimmune cells (Ivanavicius et al., 2007; Im et al., 2010; Thakur et al., 2012, 2014) features that are consistent with neuropathy. Therefore this model would be useful for assessing the analgesic potential of drugs for OA patients with neuropathic traits (Hochman et al., 2011; Duarte et al., 2014). Using *in vivo* electrophysiology, we have investigated, for the first time, the effects of ProTxII, a tarantula toxin that potently inhibits Na_v_1.7 channels with about fifteen to a hundred fold selectivity over other VGSCs (Middleton et al., 2002; Schmalhofer et al., 2008; Xiao et al., 2010), and A-803467, a selective Na_v_1.8 VGSC blocker (Jarvis et al., 2007), on the evoked activity of wide dynamic range (WDR) dorsal horn neurons in response to stimulation of the peripheral receptive field in this model of OA. The effects of ProTxII were examined after topical spinal application only because it was previously shown that ProTx-II only inhibited C-fiber action potential propagation in desheathed but not in intact nerve preparations, suggesting that the toxin could not penetrate the blood nerve barrier (Schmalhofer et al., 2008). For this reason we did not extend our ProTxII study to intraplantar and systemic routes as we did not expect that the toxin would be able to reach the channel. The effects of the selective Na_v_1.8 channel blocker A-803467 given via three different routes of administration (topical spinal, systemic and intraplantar injection) were assessed in order to shed light on the sites of action of the drug. *In vivo* electrophysiology allows for spinal nociceptive processing and central sensitization to be studied experimentally and provides information on suprathreshold responses, which are likely to equate to high levels of pain transmission as reported by patients, therefore adding to behavioral data where the analgesic effect of drugs on threshold responses are generally measured.

## Experimental procedures

Sprague–Dawley rats (Central Biological Services, University College London, UK) weighing 130–140 g at time of injection and 240–270 g at time of *in vivo* electrophysiology were employed for this study. All experimental procedures were approved by the UK Home Office and followed the guidelines under the International Association for the Study of Pain (Zimmermann, 1983).

### Induction of OA

On day 0 isoflurane anesthetized Sprague–Dawley rats received an intra-articular injection of 2–mg MIA in 25 μl of 0.9% saline through the infrapatellar ligament of the knee. Sham animals were injected with sterile 0.9% saline only. Following injection animals were allowed to recover and then re-housed in cages under a 12-h alternating light/dark cycle with *ad libitum* access to food and water.

### Assessment of pain related behavior

#### Development of mechanical and cooling hypersensitivity

Behavioral responses to stimulation of the ipsilateral hind paw were recorded once the animals had acclimatized to the testing area (Perspex cages with a wire mesh floor) for at least 30 min. Tactile hypersensitivity was tested by touching the plantar surface of the hindpaw with von Frey (vF) filaments (Touch-test TM, North Coast Medical Inc., San Jose, CA, USA) using the “up-down method” (Chaplan et al., 1994), starting with 2.0 g then ranging from 0.4 g to 15 g. Positive withdrawals were counted as biting, licking and withdrawal during or immediately following the stimulus. The strength of the vF filament was increased or decreased following a negative or positive response respectively. This up-down procedure was applied 4 times following the first change in response. Data are presented as 50% paw withdrawal threshold (PWT) for each group ± standard error of the mean (SEM). Sensitivity to cooling stimulation was assessed as the number of withdrawals out of a trial of five applications of a drop of acetone to the plantar surface of the ipsilateral hind paw. Paw withdrawal frequency (PWF) was quantified and presented as a percentage of the maximal response i.e. (number of foot withdrawals/five trials) × 100.

#### Hind-limb weight bearing

Changes in hind paw weight bearing was measured using an incapacitance tester (Linton instruments, Norfolk, UK). Animals were placed in a perspex chamber designed so that the animal is upstanding and the hindpaws rest on a separate small electronic balance so that the weight distributed on the right and left hind paw could be measured. Once the animal was settled three consecutive readings (each measured over 3 s) were recorded. The average of a total of three readings was determined for each hind limb for each rat and used for subsequent analyses. The weight bearing of the ipsilateral hindpaw to knee injection is presented as a percentage of the total weight bearing of both hind limbs.

### *In vivo* electrophysiology

Two weeks after MIA injection i*n vivo* electrophysiological studies were performed (post MIA injection days 15 and 16) as previously described (Rahman et al., 2009). Briefly, animals were anesthetized and maintained for the duration of the experiment with isoflurane (1.5–1.7%) delivered in a gaseous mix of N_2_O (66%) and O_2_ (33%). A laminectomy was performed to expose the L4–5 segments of the spinal cord. Extracellular recordings were made from ipsilateral deep dorsal horn neurons (lamina V–VI) using parylene-coated tungsten electrodes (A-M Systems, Sequim, WA, USA). All the neurons recorded in this study were WDR since they all responded to both light touch and noxious inputs (pinch and noxious heat); further all neurons responded to natural stimuli in a graded manner with coding of increasing intensity.

The evoked response to a train of 16 transcutaneous electrical stimuli (2 ms wide pulses, 0.5 Hz) applied at three times the threshold current for C-fiber activation of the dorsal horn cell. The train of electrical stimuli was delivered via stimulating needles inserted into the peripheral receptive filed, following which a post-stimulus histogram was constructed. Responses evoked by Aβ- (0–20 ms), Aδ- (20–90 ms) and C-fibers (90–350 ms) were separated and quantified on the basis of latency. Responses occurring after the C-fiber latency band were taken to be the post-discharge of the cell (350–800 ms). Two other measures of electrically evoked neuronal activity were made. The “Input” which is calculated as the number of action potentials evoked by the first stimulus (due to C-fiber activity) in the train of electrical stimuli response multiplied by 16; thus “Input” is a measure of the non-potentiated response i.e. the baseline C-fiber-evoked response which is likely a measure of afferent input and the resultant spinal neuronal response prior to central neuronal hyperexcitability evoked by subsequent stimuli. We also measured “Wind-up” which is calculated as the total number of action potentials evoked by C-fiber activity subtracting the Input. This potentiated response seen as increased neuronal activity in response to constant repetitive C-fiber stimulation is a measure of central sensitization. The center of the peripheral receptive field was also stimulated using mechanical punctate and thermal stimuli (vF filaments, 2, 8, 26 and 60 g and heat, applied with a constant water jet, 40, 45 and 48 °C) Application of each von Frey hair was separated by a minimum interval period of 5–10 s, and longer for very responsive neurons at the higher intensity range. Application of each subsequent heat stimulus was separated by a minimum period of 1 min. All natural stimuli were applied for a period of 10 s per stimulus. The mechanical and thermal natural evoked neuronal response was recorded as the number of action potentials evoked during the 10-s stimulation application period. Data were captured and analyzed by a CED 1401 interface coupled to a Pentium computer with Spike 2 software (Cambridge Electronic Design; PSTH and rate functions).

Pharmacological assessment was carried out on one neuron only per animal. The testing procedure was carried out every 20 min and consisted of a train of electrical stimuli followed by natural stimuli as described above. It should be noted that the train of electrical stimuli may be sensitizing and could enhance subsequent test responses. Thus, expression of some of the effects reported might depend upon this prior sensitization. Following three consecutive stable control trials (<10% variation for the C-fiber evoked response, and <20% variation for all other parameters) neuronal responses were averaged to give the pre-drug control values. Then either ProTxII, diluted in saline 0.9% was given via topical spinal application (0.005 and 0.05 μg/50 μl) or A-803467 diluted with 95% polyethylene glycol and 5% dimethylsulfoxide solution, via topical spinal application (10 and 50 μg/50 μl) or systemically via subcutaneous injection into the scruff of the neck (3 and 30 mg/kg) or via intraplantar injection into the ipsilateral hindpaw (10 and 50 μg/50 μl). The selection of A-803467 and ProTxII doses were based on earlier studies (Jarvis et al., 2007; McGaraughty et al., 2008; Schmalhofer et al., 2008). The effect of each dose was followed for an hour, with tests (train of electrical stimuli followed by mechanical and thermal stimulation of the peripheral receptive field, in that order) carried out at 10, 30 and 50 min before the next dose was applied cumulatively. A trend for the greatest effect was seen at either the 10- or 30-min time point (for both drugs and routes). Using this protocol the evoked responses are stable over several hours. The lack of effect of the low dose of either drug evidences this stability.

### Statistics

All statistical tests were performed on raw data using GraphPad Prism 5 (GraphPad software, La Jolla, CA, USA) and for all data a 95% confidence interval was used as a measure of statistical significance. For *in vivo* electrophysiology measures, statistical significance was tested using non-parametric Mann–Whitney test to compare two groups of data and a one-way or two-way repeated-measures analysis of variance (RM ANOVA), followed by a Bonferroni corrected paired *t*-test when simultaneously comparing more than two groups of data. Drug effects were measured as the maximum change from the averaged pre-drug control values for each dose (seen at 10-, 30- or 50-min time point) on each response per neuron (the electrophysiological unit is the number of action potentials evoked by a given stimulus). The overall effect of the drug was then expressed and presented as the mean maximal evoked neuronal response for each dose ± SEM. A one-way RM ANOVA was used to evaluate drug effects on the neuronal responses evoked by electrical and dynamic brush stimulation and a 2-way RM ANOVA was used to evaluate drug effects on the neuronal responses evoked by mechanical or heat stimulation in MIA or control rats. Behavioral data were analyzed using the Mann–Whitney test. Values of *p* < 0.05 were considered significant.

## Results

### MIA-induced behavioral hypersensitivity

A significant decrease in PWT to mechanical stimulation, a significant increase in PWF to cooling stimulation of the ipsilateral hind paw and a significant decrease in hind limb weight bearing of the side ipsilateral to MIA injection, compared with sham rats, confirmed OA pain development; PWT: MIA 2.9 ± 0.2 g vs Sham 11.5 ± 1 g, PWF: MIA 3.6 ± 0.4 lifts vs Sham 1.1 ± 0.3 lifts and weight bearing: MIA 33.7 ± 1.3% vs Sham 48.5 ± 2% at day 14 post model induction, MIA (*n* = 28) vs Sham (*n* = 27), *p* < 0.05, Mann–Whitney test (data not shown). These findings are in line with a previous study (Rahman and Dickenson, 2014).

### *In vivo* electrophysiology – evoked responses of dorsal horn neurons

**Fig. 1:**
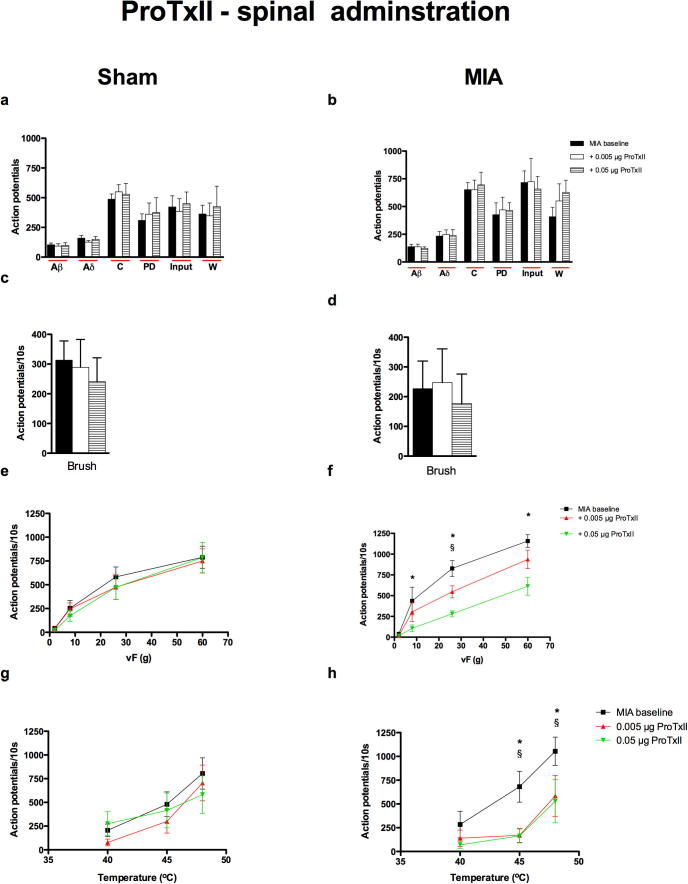
Neuronal responses evoked by vF 8–60 g and 45 and 48 °C heat stimulation and were significantly reduced by ProTxII in the MIA group only. Comparison of the effects of spinal administration of ProTxII (0.005 and 0.05 μg/50 μl) on the evoked neuronal responses to electrical (a, b), dynamic brush (c, d), mechanical punctate (e, f) and thermal stimulation (g, h) of the peripheral receptive field in sham (*n* = 8, left panel) and MIA (*n* = 7, right panel) rats. ^§^Denotes significance at 0.005 μg, and ^∗^denotes significance at 0.05 μg compared with pre-drug baseline control data, *p* < 0.05, two-way RM ANOVA with Bonferroni test for multiple paired comparisons. Values are mean ± SEM.

The effect of ProTxII or A-803467, delivered via spinal, systemic or intraplantar route, was assessed upon the evoked responses of deep dorsal horn (Lamina V–VI) neurons to electrical and natural mechanical and thermal stimulation of their peripheral receptive field. Comparison of the average baseline pre-drug responses for MIA and shams per drug and per route of administration (spinal or systemic) revealed a significantly greater C-fiber and vF 60 g evoked response in the MIA group vs sham in the ProTxII study (*p* < 0.05 Mann–Whitney test, Fig. 1 a vs b); a significantly greater response evoked by 40 °C stimulation in the MIA vs sham group in the A-803467 “systemic” study (*p* < 0.05 Mann Whitney test, Fig. 3 a vs b) and a significantly greater response evoked by vF 8 g in the MIA vs sham group in the A-803467 “intraplantar” study (*p* < 0.05 Mann–Whitney test, Fig. 4 a vs b). All other baseline neuronal responses were not significantly different between MIA and sham groups. However, this study was not powered to compare baseline neuronal responses between MIA and sham groups, therefore any differences in the average baseline neuronal responses were not further analyzed or emphasized. Although in an earlier study, where we characterized a large number of cells, we observed, on average, greater firing of neurons in response to mechanical and thermal stimulation in the MIA group, but not to electrical or brush stimuli (Rahman et al., 2009).

#### MIA-dependent antinociceptive effect of ProTxII on the mechanical and thermal evoked responses of spinal dorsal horn neurons

Topical spinal application of ProTxII did not produce any significant effects on any of the electrical stimuli, indicating a lack of effect on excitability, or brush-evoked neuronal responses in either group (Fig. 1a–d). In contrast, a clear MIA-dependent antinociceptive effect of ProTxII was observed on many of the natural mechanical punctate and thermal evoked responses. The low-threshold mechanical response evoked by vF 8 g applied to the peripheral receptive field was significantly inhibited by the top dose of ProTxII (0.05 μg) and a dose-dependent inhibition with 0.005 and 0.05 μg ProTxII was seen of the evoked neuronal response to noxious mechanical (vF 26 and 60 g) stimulation of the peripheral receptive field in the MIA group only (Fig. 1f). Similarly, ProTxII, was able to reduce the neuronal response to noxious heat (45 and 48 °C) stimulation in the MIA group only, with both doses producing an equivalent degree of significant inhibition (Fig. 1h). It has previously been shown that 0.01 mg/kg i.t. produces a plasma concentration of 3 nM (significantly lower than the IC50 for other Nav channels) (Schmalhofer et al., 2008), the doses we have used here equate to approximately 0.02–0.2 μg/kg i.t. (based on a 250 g rat) and are considerably lower. Therefore, since the dose used by Schmalhofer et al. (2008) was shown to produce Na_v_1.7-specific inhibition, it is reasonable to assume that the inhibitory effects of ProTx-II on the evoked dorsal horn neurons seen in the present study reflect a blockade of Na_v_1.7 channel activity and not other Nav channels or other off-target effects.

#### Spinal administration of A-803467 produced a marked and significant inhibition of the evoked responses of dorsal horn neurons to mechanical and thermal stimulation in the MIA group

**Fig. 2:**
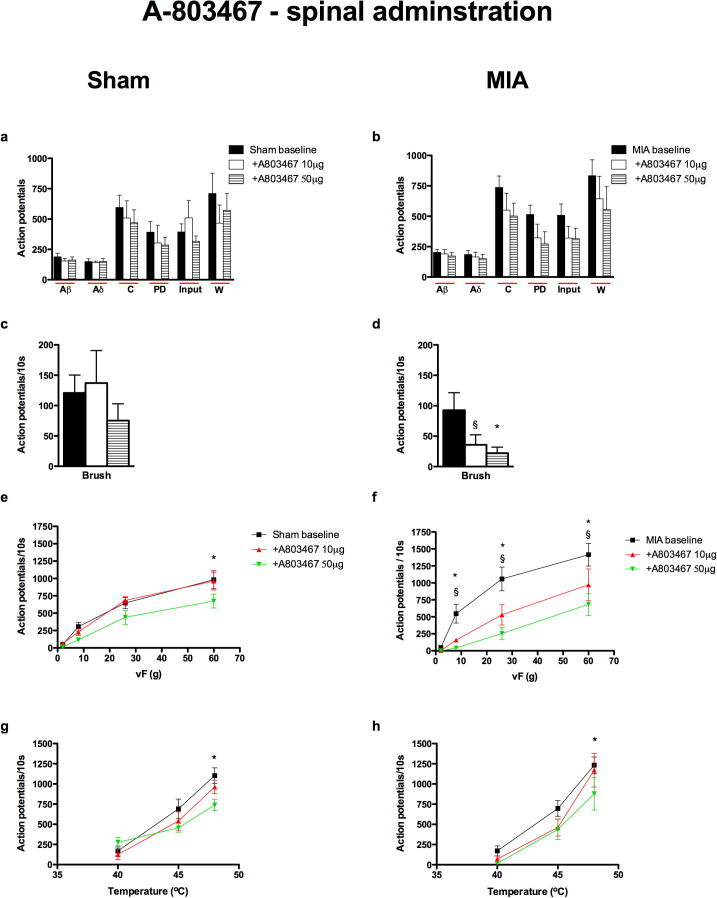
Neuronal responses evoked by brush, vF 8–60 g and 48 °C heat were significantly inhibited after spinal administration of A-803467 in the MIA group. Comparison of the effects of topical spinal administration of A-803467 (10 and 50 μg/50 μl) on the evoked neuronal responses to electrical (a, b), dynamic brush (c, d), mechanical punctate (e, f) and thermal stimulation (g, h) of the peripheral receptive field in sham (*n* = 6, left panel) and MIA (*n* = 7, right panel) rats. Asterisks and bars denote statistically significant main effect (one-way RM ANOVA). ^§^Denotes significance at 10 μg, ^∗^denotes significance at 50 μg compared with baseline control data, *p* < 0.05, two-way RM ANOVA with Bonferroni test for multiple paired comparisons. Values are mean ± SEM.

Spinal administration of A-803467 reduced some of the electrical evoked neuronal responses in both MIA and sham groups, but these effects did not reach significance (Fig. 2a, b). In complete contrast, A-803467 produced a clear MIA group-dependent inhibition of many of the mechanical- and thermal evoked neuronal responses; in particular the mechanical evoked responses in the MIA group were highly sensitive to the inhibitory effects of A-803467 (Fig. 2d, f).

In the MIA group, A-803467 inhibited the evoked neuronal response to brush stimulation, which was significant with the top dose of the drug (50 μg) (Fig. 2d). A-803467, at both doses, significantly and markedly inhibited the neuronal responses evoked by vFs 8–26 g, (Fig. 2f). The thermal evoked neuronal responses in MIA rats were also inhibited by spinal administration of A-803467, with a significant inhibition of the response evoked by 48 °C stimulation seen with the top dose (50 μg) only (Fig. 2h).

The effects of spinal administration of A-803467 on the evoked neuronal responses in the sham control rats were minimal. A-803467 produced a non-significant trend toward inhibition of the electrical C-fiber-evoked neuronal response and the PD measure of neuronal excitability. The mechanical punctate and thermal evoked neuronal responses in the sham animals were largely resistant to the effects of the drug, with the top dose of A-803467 producing a significant reduction of the evoked neuronal response to vF 60 g and 48 °C stimulation only (Fig. 2e, g).

The selective effects of A-803467 for Na_v_1.8 channels in reducing the behavioral and neuronal measures of nociception have been established in other models of chronic pain (Jarvis et al., 2007; McGaraughty et al., 2008). The doses of spinal A-803467 employed in the present study equate to 28–140 nmol/50 μl which are considerably lower than those used by McGaraughty et al., 2008 (McGaraughty et al., 2008.) Therefore it is likely that the inhibitory effects of spinal administration of A-803467 seen in the present study reflect a selective blockade of Na_v_1.8 channels and not other Nav channels.

#### MIA-dependent antinociceptive effect of mechanical and thermal evoked responses of dorsal horn neurons following systemic administration of A-803467

**Fig. 3:**
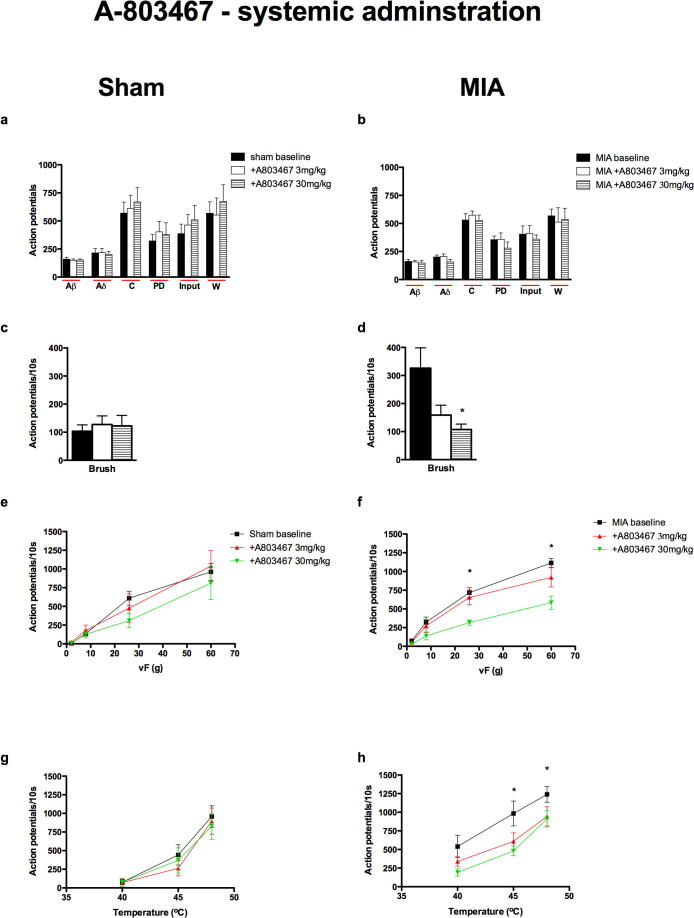
Neuronal responses evoked by brush, vF 26 and 60 g and 40 and 45 °C heat were significantly inhibited after systemic administration of A-803467 in the MIA group only. Comparison of the effects of systemic administration of A-803467 (3 and 30 mg/kg) on the evoked neuronal responses to electrical (a, b), dynamic brush (c, d), mechanical punctate (e, f) and thermal stimulation (g, h) of the peripheral receptive field in sham (*n* = 7, left panel) and MIA (*n* = 7, right panel) rats. ^∗^Denotes significance at 30 mg/kg compared with baseline control data, *p* < 0.05, two-way RM ANOVA with Bonferroni test for multiple paired comparisons. Values are mean ± SEM.

The systemic doses of A-803467 used in the present study are in line with those used in earlier studies in different models of chronic pain (Jarvis et al., 2007; McGaraughty et al., 2008). The electrical evoked neuronal responses were not significantly affected by systemic administration of A-803467 (3 and 30 mg/kg) to either group (Fig. 3a, b), in line with the lack of effect of the drug on these neuronal measures after spinal application. A significant inhibition of the mechanical brush and vFs 26–60 g and thermal, 40–48 °C, evoked neuronal responses was seen in the MIA-treated group only, suggesting an MIA-dependent anti-hyperalgesic action of 30 mg/kg A-803467 via sub cutaneous administration (Fig. 3f, h). In comparison these doses of A-803467 had no significant effect on any neuronal measure in the sham group.

#### Intraplantar administration of A-803467 inhibited the electrical, mechanical and thermal evoked responses of dorsal horn neurons in the MIA group

**Fig. 4:**
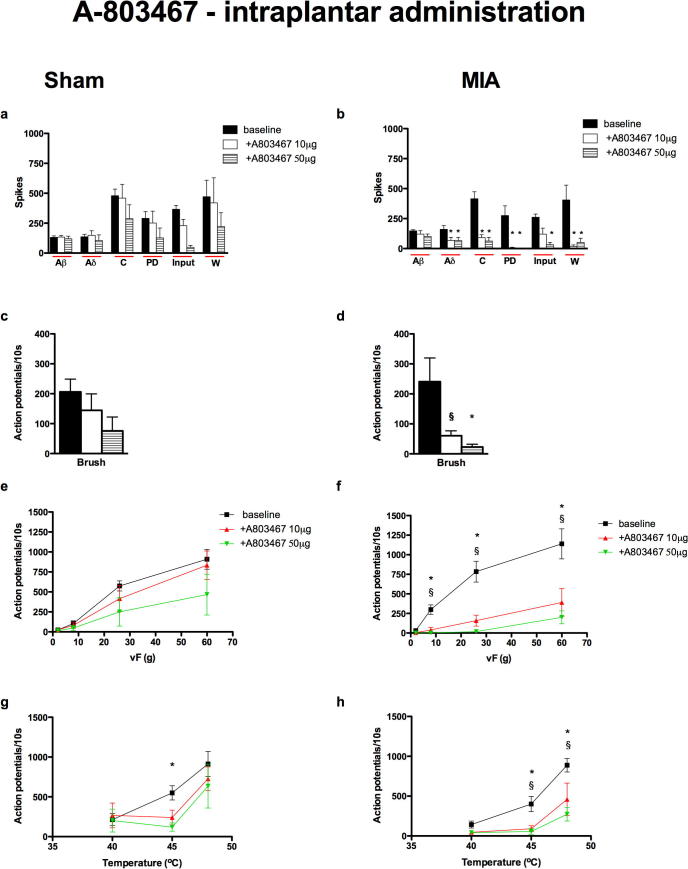
Intraplantar administration of A-80347 significantly reduced the Aδ-, C-fiber, post-discharge, Input, Wind-up, brush, vF 8–60 g and 45 and 48 °C heat evoked neuronal responses in the MIA group. Comparison of the effects of intraplantar administration of A-803467 (10 and 50 μg/50 μl) on the evoked neuronal responses to electrical (a, b), dynamic brush (c, d), mechanical punctate (e, f) and thermal stimulation (g, h) of the peripheral receptive field in sham (*n* = 6, left panel) and MIA (*n* = 7, right panel) rats. ^§^Denotes significance at 10 μg, ^∗^denotes significance at 50 μg compared with baseline control data, *p* < 0.05, one-way or two-way RM ANOVA with Bonferroni test for multiple paired comparisons. Values are mean ± SEM.

Intraplantar administration of A-803467 (10 μg and 50 μg/50 μl) produced a marked and significant inhibition of nearly all the evoked neuronal responses in the MIA group. Both doses of the drug inhibited the electrically evoked Aδ and C-fiber responses as well as the neuronal excitability measures of post-discharge, Input and Wind-up, indicative of this peripheral route allowing attenuation of nerve excitability or propagation. The mean response evoked by dynamic brush, vFs 8–60 g and 45–48 °C heat was significantly inhibited by both doses of A-803467 (Fig. 4b, d, f, h). In contrast, in the sham control group, the top dose of A-803467 was effective in reducing the Input and neuronal response evoked by 45 °C heat stimulation only (Fig. 4a, g).

The doses of A-803467 given by intraplantar injection in the present study equate to 28 and 140 nmol/50 μl and are lower than the dose used by others, where a significant and selective inhibitory effect of A-803467 on the evoked responses of WDR neurons was seen following injection of 300 nmol/50 μl into the hind paw receptive field (McGaraughty et al., 2008). Therefore the inhibitory effects of A-803467 following intraplantar injection seen in the present study likely reflect a selective blockade of Na_v_1.8 channels and not other Nav channels.

## Discussion

OA is a progressive and degenerative disease of the whole joint and typically includes a destruction and degradation of the articular cartilage, subchondral bone, synovial lining and connective tissues (Vincent and Watt, 2014). In this study we have used the MIA model of OA. This is a chemically induced, rapidly progressive model that is well described in the rat especially in terms of its disease pathology (Guzman et al., 2003) and mirrors many aspects of the human condition, and has so far proved useful for the understanding of osteoarthritic pain mechanisms (Zhang et al., 2013). In addition this dose of MIA has been shown to produce OA associated with markers of neuropathy (Ivanavicius et al., 2007; Im et al., 2010; Thakur et al., 2012, 2014) and therefore may be indicative of those patients with advanced disease that display an additional neuropathic pain phenotype (Hochman et al., 2011; Duarte et al., 2014). Knee joint pathology was not assessed here, however we have previously demonstrated cartilage loss following injection of 2 mg of MIA, which is characteristic of human OA, (Thakur et al., 2012) as have others (Fernihough et al., 2004; Pomonis et al., 2005; Im et al., 2010), also MIA injection produced hypersensitivity to mechanical and cooling stimulation of the ipsilateral hind paw and a decrease in hind limb weight bearing of the injected side confirming OA pain development (Vincent et al., 2012; Malfait et al., 2013).

The behavioral hypersensitivity to stimulation of the ipsilateral hind paw, i.e. the referred receptive field area, reflects secondary hyperalgesia, which is indicative of central sensitization. Pain symptoms elicited by various activities such as bending or walking in patients with knee OA are largely associated with the area surrounding the affected joint, but referred pain and tenderness also occurs implicating mechanisms of central sensitisation contributing to their pain (Farrell et al., 2000; Bajaj et al., 2001; Gwilym et al., 2009; Graven-Nielsen and Arendt-Nielsen, 2010; Aranda-Villalobos et al., 2013), and a direct link between the level of sensitization in referred areas and clinical pain intensity experienced by OA patients has been shown (Arendt-Nielsen et al., 2010). Therefore the data presented here provide for an electrophysiological and behavioral correlate for the spread of sensitization seen in OA patients and allows for the study of spinal nociceptive processing and central sensitization mechanisms.

Referred pain is dependent not only on central hyperexcitability but also on input from the periphery (Laursen et al., 1997; Graven-Nielsen and Arendt-Nielsen, 2010; Baron et al., 2013), therefore there is a logical basis for targeting this, and central neuronal excitability, by blocking sodium channel function, thus reducing action potential generation and transmission. In this study, we assessed the effects of two different sodium channel blockers, ProTxII and A-803467, which block Na_v_1.7 and Na_v_1.8 channels respectively (Middleton et al., 2002; Jarvis et al., 2007; Schmalhofer et al., 2008; Xiao et al., 2010), on the evoked responses of WDR neurons located in the deep dorsal horn of the spinal cord. Our findings show that both drugs, via different routes of administration, significantly inhibited neuronal activity in the MIA group, suggesting a greater contribution of Na_v_1.7 and 1.8 channel activity in mediating nociceptive transmission in the arthritic condition. This is consistent with observations of increased expression of Na_v_1.7 and 1.8 in dorsal root ganglia (DRG) neurons during OA (Strickland et al., 2008). Further, both drugs produced “selective” inhibition of neuronal responses in the pathological condition. This is key, since it means that both drugs would allow physiological transmission yet attenuate abnormal pathophysiological transmission.

Importantly, the *in vivo* electrophysiological technique we have used not only enables measurement of low threshold innocuous evoked neuronal activity, but also suprathreshold evoked neuronal responses. Many pain studies evaluate around a nociceptive threshold, whereas clinical pain is almost always more severe, thus our *in vivo* electrophysiological recordings provide a correlate for the high-intensity pain scores reported by patients, and therefore adds to the findings from behavioral approaches.

The Na_v_1.7 channel is expressed in sensory and sympathetic neurons and olfactory epithelial cells (Black et al., 1996, 2012; Cummins and Waxman, 1997). Several lines of evidence have firmly placed this channel in pain pathways, with compelling evidence from genetic studies of rare human pain states (see Refs. Dib-Hajj et al., 2013). Indeed a mutation in the encoding gene for Na_v_1.7 (SCN9A) has been associated with a greater pain score in OA patients (Reimann et al., 2010), but see (Valdes et al., 2011). Therefore targeting and modulating aberrant activity of Na_v_1.7 channel activity should prove useful for pain associated with OA. Our *in vivo* electrophysiological data support this hypothesis since ProTxII significantly reduced low- and high-intensity mechanical evoked neuronal responses, and complements *in vivo* electrophysiological data from knockout (KO) mice where Na_v_1.7 channels deleted from all sensory neurons produced a reduction of mechanical evoked neuronal responses (Minett et al., 2012). Taken together these findings confirm the requirement of Na_v_1.7 activity for mechanical evoked neuronal responses. Interestingly, ProTxII also produced marked and significant inhibitions of the noxious heat-evoked neuronal response in the MIA group. This also aligns with data from mutant mice studies. Mice with a conditional KO of Na_v_1.7 (from a subset of sensory neurons that expresses Na_v_1.8) do not display signs of hypersensitivity to heat stimuli after undergoing burn model injury or in the CFA model of inflammatory pain (Shields et al., 2012b), and significant reductions in the electrophysiological responses of spinal neurons to noxious heat were seen in mice lacking Na_v_1.7 in all sensory neurons (Minett et al., 2012), although in the same study the behavioral response to noxious heat in the Hargreaves and hotplates tests were only attenuated in mice where Na_v_1.7 was deleted in all sensory and sympathetic neurons. Since ProTxII does not discriminate between neuronal subpopulations our data complement the findings of Shields et al. (2012b) and Minett et al. (2012) and verify a role for Na_v_1.7-mediating noxious thermal hyperalgesia.

In contrast to its inhibitory effects on the natural evoked neuronal responses in the MIA group, ProTxII did not affect the neuronal responses induced by electrical stimulation. This may be because the barrage of activity induced by the train of 16 electrical stimuli maybe too great for the drug at this dose to overcome. It is also possible that natural mechanical- and thermal evoked neuronal responses are more sensitive to the inhibitory effects of the drug. However the most likely explanation is that under these conditions the channel blockers prevent the transduction and/or transmission from sensory receptors without global effects on peripheral nerve excitability. However in the presence of these drugs physiological evoked responses of spinal sensory neurons are reduced.

Expression of Na_v_1.7 channels were originally proposed to be restricted to the peripheral nervous system, however a recent study has demonstrated expression on pre-terminal sensory axons and terminals of DRG neurons in the dorsal horn (Black et al., 2012) and the marked inhibitory effects of spinal application of ProTxII seen in the present study would agree with a central spinal location for these channels. It was not possible to ascertain whether or not a similar MIA state-dependent effect of ProTxII would be seen via different administrative routes as it has been reported that the drug is unable to permeate the blood nerve barrier (Schmalhofer et al., 2008), hence precluding assessment of its effects via systemic or local routes of administration. Nonetheless, the findings from the present study indicate an increased sensitivity of Na_v_1.7 channels, at least in spinal nociceptive pathways and possibly DRG, in the arthritic condition, suggesting that Na_v_1.7 channels located on central terminals within the dorsal horn and/or DRG are functionally important under pathological conditions.

There is a large body of evidence linking Na_v_1.8 channel activity with the initiation and maintenance of chronic pain (Amir et al., 2006; Eijkelkamp et al., 2012), crucially this includes evidence from human genetic data where gain of function mutations in neuropathic patients demonstrates a link between Na_v_1.8 and the human pain experience (Faber et al., 2012). As increases in Na_v_1.8 expression have been reported under persistent inflammatory pain conditions (Tanaka et al., 1998; Amaya et al., 2000; Coggeshall et al., 2004; Villarreal et al., 2005; Strickland et al., 2008; Belkouch et al., 2014) but see (Shields et al., 2012a), it would be reasonable to propose that reducing Na_v_1.8 function, alongside improvement in the bioavailability and tolerability of small molecule Na_v_1.8 blockers, hold promise for their analgesic potential in treating chronic inflammatory states such as OA pain (Scanio et al., 2010; Zhang et al., 2010b). Indeed A-803467 has been shown to significantly attenuate hypersensitive behavior in a variety of animal models of inflammatory pain (Jarvis et al., 2007) including OA pain (Schuelert and McDougall, 2012). This latter study demonstrated an inhibitory effect following intra articular injection of A-803467 on the mechanosensitivity of joint afferents and a reduction in joint pain behavior and secondary allodynia, confirming an important role for Na_v_1.8 channels in OA pain, but they did not investigate the effects of the drug in sham controls. Our findings show that, regardless of route of administration, A-803467 produced a significant and preferential inhibition of neuronal activity in the MIA group only, suggestive of a generalized state of abnormal sensitivity within the area of referred pain. In comparison A-803467, via all three routes of administration, produced minor inhibitions of neuronal activity in the sham group. Therefore our findings add to the literature since not only do we show that A-803467 produced a marked antinociceptive effect of the drug in the MIA group, but the differential effect of the drug in the two groups suggests an alteration in functional activity of Na_v_1.8 channels at both peripheral (nerve and/or DRG) and central spinal locations in the arthritic condition. Furthermore our findings provide a neuronal correlate for the reduction of secondary allodynia observed by Schuelert and McDougall (2012) since A-803467 reduced the evoked neuronal responses to mechanical stimulation of the hind paw.

Interestingly, A-803467, via all three routes of administration, significantly inhibited the dynamic brush-evoked response in the MIA group only. The expression of Na_v_1.8 VGSC was first thought to be restricted to small diameter unmyelinated nociceptive neurons, however recent immunohistochemical data suggest that Na_v_1.8 is not exclusive to nociceptors, but is, in fact, expressed in relatively high levels (about 40%) of A-fibers and also present on C-low-threshold mechanoreceptors (C-LTMs) (Shields et al., 2012a). Indeed it has been shown that mechanical hypersensitivity requires C-LTMS (Seal et al., 2009). Therefore it is not unexpected that A-803467 was able to reduce the brush-evoked neuronal response. Additionally, alterations in the electrophysiological properties of Aβ-fiber low-threshold mechanoreceptors have been reported in a surgically induced model of OA (Wu and Henry, 2010). This may reflect a change in sodium currents in these afferents and could underlie the preferential effect of A-803467 on the brush-evoked neuronal responses seen in MIA rats in the present study. Further, a recent study also reported a functional up-regulation of Na_v_1.8 channels in Aβ fibers in a model of chronic inflammation (Belkouch et al., 2014), thus it is possible that a similar up-regulation also occurs in this model of knee OA which may contribute to the MIA-dependent inhibitory effect of A-803467 on the brush-evoked neuronal response. Alternatively, A-803467 shows a preferential affinity for inactivated channels (Jarvis et al., 2007), it is possible that a greater proportion of Na_v_1.8 channels are in this conformational state in the MIA rats, since the inactivation state of VGSCs can be induced by repeated neuronal firing and/or under conditions of sustained membrane depolarization which is probable for OA as an increased incidence of spontaneous activity and enhanced responsiveness of joint nociceptors and dorsal horn neurons has been reported (Schuelert and McDougall, 2006, 2008, 2009; McDougall et al., 2009; Rahman et al., 2009; Sagar et al., 2010; Kelly et al., 2012, 2015; Bullock et al., 2014). Taken together, our findings highlight further the potential of Na_v_1.8 as an analgesic target and suggest that blocking these channels could be effective against tactile allodynia in arthritic pain.

Spinal and systemic administration of A-803467 did not affect the neuronal responses induced by electrical stimulation, compared with the effects seen on the responses induced by natural stimuli in the MIA group. As already mentioned, this could be due to the barrage of activity induced by the train of 16 electrical stimuli being too great for the drug, at the doses given, to overcome. Again, the most likely explanation is that under these conditions the channel blockers prevent the transduction and/or transmission from sensory receptors without global effects on nerve excitability. By contrast local peripheral administration of A-803467 produced the most profound reductions in neuronal activity in the MIA group including significant inhibition of the electrical evoked responses suggesting that this local high dose alters nerve excitability, again, this may be due to an increased peripheral expression of Na_v_1.8 channels or due to a greater proportion of these channels being in the inactivated state in the arthritic animals.

Interestingly, the data also suggest that the Na_v_1.8 channels located at peripheral nerve fiber endings in distal areas play an important role in regulating nociceptive transmission in the arthritic condition. For a significant proportion of OA patients, it is likely that a large peripheral drive initiates and maintains OA pain, (Kirwan et al., 1994; Creamer et al., 1996; Ethgen et al., 2004) therefore for those OA pain patients, local administration or systemic administration of a peripherally restricted version of a Na_v_1.8 blocker would be an appropriate treatment option, as well as the obvious potential for reduced CNS side effects.

## Conclusion

The therapeutic utility of sodium channel blockers are not traditionally recommended for the treatment of OA pain, but given the large peripheral drive that follows the development of OA alongside the evidence for abnormal firing in peripheral and central neurons in the arthritic condition, implicates a key role for VGSCs in mediating OA pain. Our findings support this hypothesis since the action of ProTxII and A-803467, to favor an inhibition of neuronal responses evoked by both low-threshold and suprathreshold stimuli in the MIA group suggests for a greater contribution of these channels, at peripheral and central locations, to the arthritic pain condition. Furthermore our protocol models secondary hyperalgesia; blocking Na_v_1.7 and 1.8 channel activity reduced neuronal activity evoked from a referred site (hind paw). This is key because the level of sensitization at sites distal to the diseased joint has been directly linked to the level of pain experienced by OA patients (Arendt-Nielsen et al., 2010). Therefore assessment of the effect of drugs on both primary and secondary hyperalgesia will be important for the development of future medicines.

The model of MIA used in the present study exhibits features of neuropathy, therefore drugs designed to block VGSCs may have greater therapeutic use in OA patients with neuropathic traits who are refractory to classical medications such as NSAIDs. Certainly a better understanding of the role of Na_v_1.7 and 1.8 in mediating osteoarthritic pain will aid the development of future analgesics and the findings from the present study suggest that modulating the activity of Na_v_1.7 and 1.8 VGSCs at peripheral and/or central spinal locations could prove worthwhile for the treatment of OA pain and merits further clinical investigation.

## Competing interests

The authors declare that they have no competing interests.

## Authors’ contributions

WR conceived, designed and performed the experiments, analyzed the data and wrote the manuscript. AHD conceived and designed the experiments and helped write the manuscript. All authors read and approved the final manuscript.
